# The Effect of Text Messaging on the Postoperative Pain Experience in Pediatric Patients Undergoing Thoracic Surgery: Randomized Controlled Trial

**DOI:** 10.2196/81806

**Published:** 2026-03-24

**Authors:** Yun Shi, Qianqiu Wang, Aihua Liu, Li Jiang

**Affiliations:** 1 Department of Anesthesiology, Children's Hospital of Fudan University, 399 Wanyuan Road, Minhang District, Shanghai, 201102, China, 86 13817941882

**Keywords:** SMS, thoracic surgery, pain, pain self-efficacy, pediatric, text messaging

## Abstract

**Background:**

Inadequately controlled postoperative pain continues to pose a significant clinical challenge in pediatric patients undergoing thoracic surgery.

**Objective:**

This randomized controlled study aimed to investigate the effectiveness of SMS-based educational support for postoperative pain management on patients’ pain experience.

**Methods:**

A total of 100 pediatric patients undergoing thoracic surgery were enrolled between December 2, 2023, and January 28, 2025. Patients in the intervention group (group 1) received structured postoperative pain management education via SMS text messages, whereas those in the control group (group 2) received standard oral education. Pain intensity and pain-related interference were assessed using the Brief Pain Inventory, and self-efficacy was measured using the Pain Self-Efficacy Scale.

**Results:**

The number of patients with moderate-to-severe average pain was significantly lower in group 1 than in group 2 (n=19, 18% vs n=19, 38%; *P*=.04), and group 1 had significantly higher patient self-efficacy scores (mean 29.3, SD 7.5 vs mean 25.2, SD 8.7*; P*=.01). Least pain scores were lower in group 1 compared with group 2 across all 3 consecutive postoperative days (PODs): POD1 (mean 2.8, SD 0.8 vs mean 3.3, SD 0.7; *P*<.001), POD2 (mean 2.2, SD 0.8 vs mean 2.7, SD 0.5*; P*<.001), and POD3 (mean 1.7, SD 0.7 vs mean2.2, SD 0.4; *P*<.001). Similarly, average pain intensity was lower in group 1 compared with group 2 across all 3 consecutive PODs: POD1 (mean 3.7, SD 0.9 vs mean 4.7, SD 0.6; *P*<.001); POD2 (mean 3.3, SD 0.8 vs mean 3.6, SD 0.5; *P*=.01); and POD3 (mean 2.5, SD 0.8 vs mean 3.1, SD 0.5; *P*<.001). General activity was significantly less affected in group 1 on POD 1 (mean 4.3, SD 1.0 vs mean 5.0, SD 1.5; *P*=.004) and POD 2 (mean 3.1, SD 0.7 vs mean 3.7, SD 1.3; *P*=.009).

**Conclusions:**

The use of an SMS-based educational intervention significantly improved the postoperative pain experience of pediatric patients undergoing thoracic surgery. Further research is needed to clarify its impact on clinical outcomes and to better understand the mechanisms underlying improved pain management.

## Introduction

Thoracic surgery is typically associated with substantial postoperative pain. To optimize analgesia, a multimodal pain management strategy is widely adopted, combining nonsteroidal anti-inflammatory drugs such as ibuprofen [1] and paracetamol [2], regional anesthesia [3], and a patient-controlled analgesia pump (PCAP) [4]. Within this framework, both patients and caregivers are encouraged to participate actively in postoperative pain management, including pain assessment and analgesic administration via a PCAP. Despite these efforts, pain following thoracic surgery often remains inadequately controlled. Previous studies have reported that approximately 54% of pediatric patients experience moderate-to-severe pain on the day of surgery, and both patients and parents frequently express dissatisfaction with postoperative pain management [5]. Pain has a profound impact on the quality of postoperative recovery. As a negative and inherently subjective experience, pain is perceived directly by children. Inadequate analgesia compromises effective breathing, coughing, and moving, thereby increasing the risk of respiratory complications and delaying postoperative recovery [6,7]. Furthermore, insufficient control of acute postoperative pain has been associated with an elevated risk of developing chronic pain after discharge [8]. Several factors may contribute to suboptimal pain control in children, even in the presence of a PCAP. These include difficulties in accurately assessing pain severity, uncertainty regarding the appropriate timing of analgesic administration, challenges in adhering to medication regimens, and concerns regarding potential side effects of analgesics [9-11]. Collectively, these barriers may be attributed to insufficient educational information and/or ineffective educational support for patients and their caregivers [12].

With the rapid development of mobile technologies, internet-based medical support has emerged as an increasingly prominent component of health care delivery. The effectiveness of digital health interventions in enhancing self-management among pediatric patients with chronic diseases has been well documented [13,14]. These approaches emphasize patient-centered self-care, with health care professionals facilitating self-management by providing informational, emotional, and social support. Recently, emerging studies have investigated the use of SMS text messaging in pediatric pain management, with most focusing on pain assessment and monitoring. The feasibility and acceptability of SMS-based interventions have been widely demonstrated, as reflected by high response rates [15-17], strong caregiver acceptance [18], improved medication adherence, and reduced anxiety levels [19]. Several studies suggest that participants demonstrate improved pain coping and reduced anxiety and depression [20]. However, the effect of SMS on pain outcomes, particularly pain severity, remains unclear. More broadly, evidence regarding the impact of digital health tools, including mobile apps and other implementation strategies, on pediatric pain severity is limited and inconsistent. Some studies have reported improved pain self-management among pediatric patients [16,21-23], whereas others have found no significant benefit in pain control [20,24]. Notably, one study reported greater pain severity among patients in the intervention group [25]. Consequently, the use of SMS for postoperative pain management in pediatric populations remains in its early stages. Further research is warranted to evaluate the effects of SMS-based interventions on postoperative pain outcomes in pediatric populations.

In this prospective randomized controlled trial, an intervention group (group 1), which received SMS-based educational support for postoperative pain management, was compared with a control group (group 2), which received conventional educational support, to evaluate the effectiveness of SMS in helping manage the postoperative pain experience in pediatric patients undergoing thoracic surgery. The hypothesis was that pediatric patients receiving SMS-based educational support would demonstrate improved pain coping and reduced postoperative pain intensity.

## Methods

### Study Participants

Pediatric patients who underwent elective thoracic surgery at the Children’s Hospital of Fudan University were consecutively recruited for this randomized controlled trial. Eligible patients were (1) aged 7 to 18 years, (2) classified as American Society of Anesthesiologists (ASA) physical status I or II, (3) expected to receive a combination of a preoperative paravertebral block and a PCAP for postoperative pain management, and (4) had access to a smartphone (iOS or Android). Exclusion criteria included (1) chronic pain (defined as pain persisting or recurring for more than 3 months), (2) a history of psychiatric disorders, (3) planned video-assisted thoracoscopic surgery, (4) inability to assess pain severity using a numeric rating scale (NRS), (5) communication difficulties, (6) any prior surgeries, (7) postoperative transfer to the intensive care unit (ICU), or (8) refusal to participate.

### Study Design

Patients who met the inclusion criteria were consecutively enrolled during a preoperative visit. Participants were assigned identification numbers in the order of recruitment. An independent statistician generated the random allocation sequence using SPSS software (version 20; IBM Corp). The allocation sequence was placed in sealed envelopes, which were opened only after participant enrollment. Separate investigators were responsible for patient enrollment and follow-up. All patients underwent surgery under general anesthesia. Due to the study design, participants were not blinded to the intervention in either group, but they did not have information about each other. The statistician remained blinded to group allocation.

### Procedure

Participants received comprehensive educational information on postoperative pain management. The information covered the cause of postoperative pain, advantages and disadvantages of PCAP, indications for PCAP, operational principles of PCAP, self-assessment of pain, pain management goals, proper use of PCAP, nonpharmacological pain relief methods, scheduled medication reminders, and guidance on early postoperative mobilization. Group 1 received information through a combination of text, illustrative images (eg, pain assessment scales), and instructional videos (eg, demonstrating how to operate the PCAP). Concise (Multimedia Appendix 1) and detailed (Multimedia Appendix 2) versions of the educational content were provided to facilitate participant understanding. The concise version emphasized practical operation and workflow, whereas the detailed version provided more comprehensive explanations to support a deeper understanding. The information was sent out manually on the day prior to surgery. Group 2 received conventional oral education support that provided the same information. All participants were instructed on the importance of self-monitoring and managing postoperative pain to promote engagement with pain self-management. Participants were encouraged to actively assess and manage their own pain, with parental assistance permitted as needed. They were advised to contact health care providers whenever necessary. However, participants in group 1 were not allowed to communicate with the investigators via SMS.

### Outcomes and Measures

All patients underwent surgery under general anesthesia. A preoperative paravertebral block was performed, and a PCAP was initiated upon discharge from the postoperative anesthesia care unit. Demographic data, including age, sex, place of residence, and parental educational level, were collected prior to surgery. Patients were followed for the first 3 postoperative days (PODs) by a research nurse at 24, 48, and 72 hours after surgery. At each time point, the cumulative dose of hydromorphone administered via PCAP was retrieved from the system and recorded. If pain was inadequately controlled, additional rescue analgesic doses were permitted, and dosages were verified through patient medical records. As hydromorphone doses were calculated according to body weight, the cumulative dosage was analyzed and compared on a per-day, per–body weight basis.

Patient-reported pain intensity and pain interference were assessed over the 3 consecutive PODs using the Brief Pain Inventory (BPI) questionnaire. The BPI, recommended by the National Cancer Institute, is used to evaluate pain severity using an NRS ranging from 0 (no pain) to 10 (worst pain), capturing the highest, lowest, and average pain experienced. It also assesses the impact of pain on daily functioning, including activities, mood, sleep, interpersonal relationships, and enjoyment of life. Consequently, the BPI is widely used in clinical practice [26] and research to provide detailed information on pain experience [27-29]. Pain self-efficacy refers to a patient’s belief and confidence regarding their ability to effectively manage pain and attain desired outcomes. Pain self-efficacy is recognized as an important resilience factor for pain, particularly in children and adolescents [30]. The Pain Self-Efficacy Scale (PSES) [31] was used to evaluate the patient’s confidence in managing pain. The scale consists of 10 items, each scored from 1 to 6, and was administered both before surgery and on POD3. Patient satisfaction with postoperative pain management was evaluated on POD3 using an NRS (0=not satisfied at all, 10=very satisfied). Analgesic-related complications—including postoperative nausea and vomiting, urine retention, constipation, severe sedation, and respiratory depression—were also recorded.

The primary outcome of the study was the incidence of moderate-to-severe average pain on POD1, defined as an NRS score of 4 or greater. Secondary outcomes included cumulative hydromorphone consumption, pain experiences and their impact on daily life, pain self-efficacy, analgesic-related complications, and patient satisfaction.

### Sample Size Estimation

A pilot study conducted at the Children’s Hospital of Fudan University reported that the incidence of moderate-to-severe average pain on POD1 was 39.5%. A clinically meaningful reduction was defined as a one-third decrease in this incidence with the implementation of SMS for postoperative pain management. Assuming 80% power and a significance level of .05, the minimum required sample size was calculated as 45 patients per group. To account for an anticipated 10% loss to follow-up, the sample size was increased to 50 patients per group.

### Data Analysis

Only participants who completed the entire questionnaire were included in the analysis. Data were analyzed using GraphPad Prism (version 9; Graphpad Corp) and SPSS software (version 20; IBM Corp). Continuous variables were tested for normality. Normally distributed data were expressed as means with SDs and compared using independent *t* tests. Conversely, nonnormally distributed data were expressed as medians with IQRs and compared using the Mann-Whitney *U* test. Categorical data were expressed as numbers and percentages and analyzed using the chi-square test. Univariate association between the incidence of moderate-to-severe average pain and PSES score was analyzed using the Poisson correlation.

### Ethical Considerations

The study was conducted at the Children’s Hospital of Fudan University and approved by the research ethics board (202328; approval date June 20, 2023; chairperson Li-Ling Qian). The study was registered at Chictr.org.cn (ChiCTR2300076554). Eligible patients who met the inclusion criteria and their parents were approached in a separate interview room 1 day before surgery. Detailed explanations of the study objectives, methods, potential benefits, and risks were provided. Participants were informed that their medical data would be reported publicly, but no personally identifiable information would be included. They were assured that participation would not affect the quality of care and that withdrawal from the study was permitted at any time. All the responders volunteered to participate free of charge. Signed informed consent was obtained from the parents of participants who agreed to participate in the study.

## Results

### Study Population

**Figure 1.**
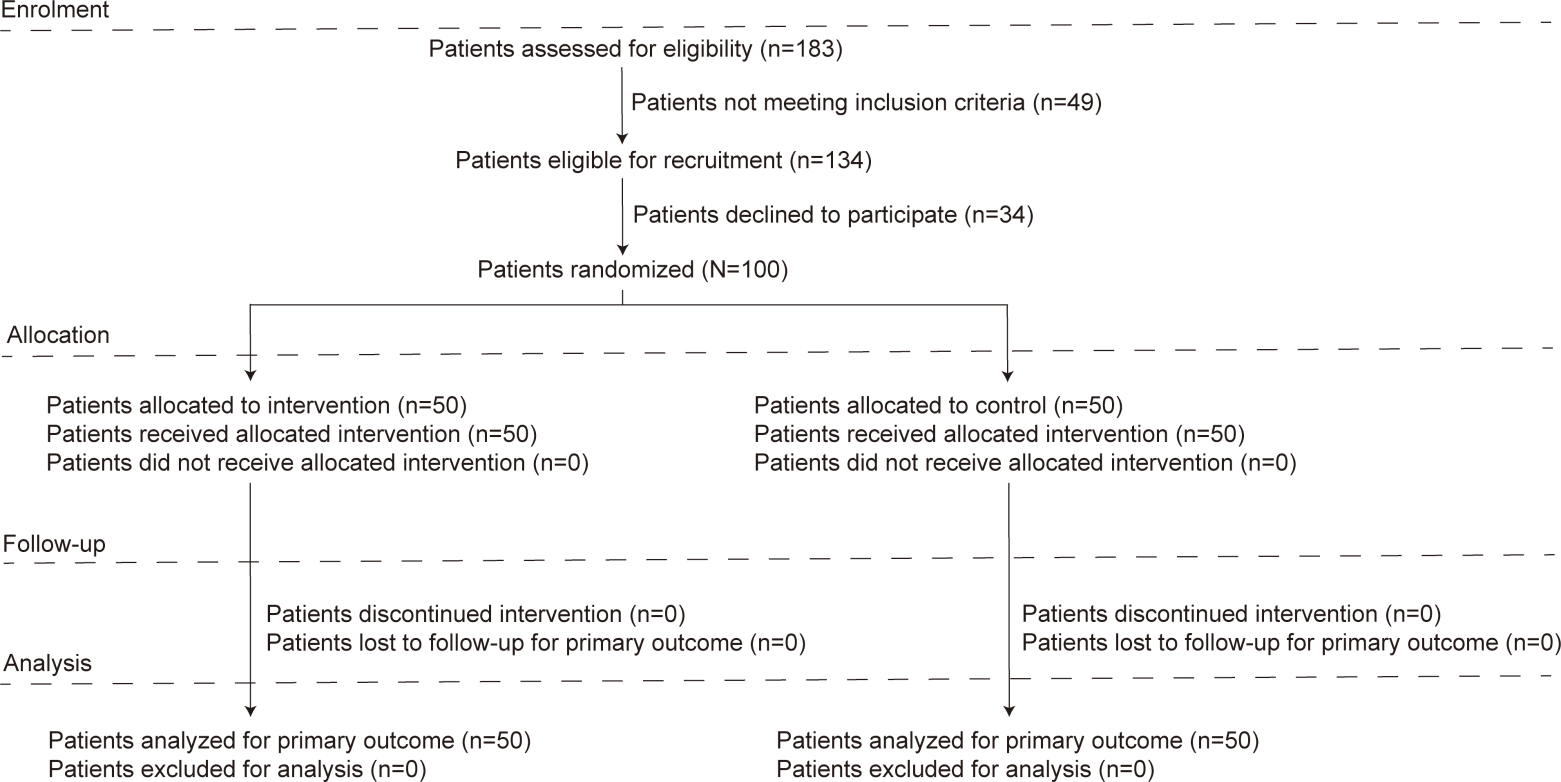
Study flow diagram.

From December 2, 2023, to January 28, 2025, a total of 183 patients were assessed for eligibility. Of these, 134 patients met the inclusion criteria, and 100 (76.4%) agreed to participate. None of the patients were excluded from the analysis owing to missing data or loss to follow-up (Figure 1). No significant differences were observed between the groups in terms of age, sex, weight, diagnosis, and parental educational level (Table 1).

**Table 1.** Participant characteristics (N=100).

		Intervention group (n=50)	Control group (n=50)	P value
Age (y), mean (SD)		10.7 (2.8)	11.1 (2.9)	.48
Gender, n (%)				>.99
	Male	33 (66)	32 (64)	
	Female	17 (34)	18 (36)	
Weight (kg), mean (SD)		39.9 (11.7)	40.1 (14.2)	.94
Parent education, n (%)				.61
	Middle school	8 (16)	10 (20)	
	High school	10 (20)	12 (24)	
	Associate degree	18 (36)	15 (30)	
	Undergraduate degree	12 (24)	13 (26)	
	Master’s degree and above	2 (4)	0 (0)	
Diagnosis, n (%)				.17
	Rib lesions	3 (6)	2 (4)	
	Diaphragmatic lesions	3 (6)	2 (4)	
	Pectus excavatum/carinatum	12 (24)	24 (48)	
	Mediastinal mass	16 (32)	12 (24)	
	Pulmonary lesions	16 (32)	10 (20)	
History of surgery, n (%)				.68
	Yes	22 (44)	19 (38)	
	No	28 (56)	31 (62)	
Experience with PCAP, n (%)				.15
	Yes	15 (30)	8 (16)	
	No	35 (70)	42 (84)	
PCAP operator, n (%)				.69
	Patient	0 (0)	0 (0)	
	Parent	22 (44)	25 (50)	
	Both	28 (56)	25 (50)	

a PCAP: patient-controlled analgesia pump.

### Primary Outcome

The incidence of moderate-to-severe average pain on POD1 was significantly lower in group 1 than in group 2 (n=9, 18% vs n=19, 38%; *P*=.04), whereas no difference was detected between the groups on POD2 and POD3 (Table 2).

**Table 2.** Pain experience among pediatric patients after thoracic surgery (N=100).

	Postoperative day 1			Postoperative day 2			Postoperative day 3		
	Group 1 (n=50)	Group 2 (n=50)	*P* value	Group 1 (n=50)	Group 2 (n=50)	*P* value	Group 1 (n=50)	Group 2 (n=50)	*P* value
Primary outcome, n (%)									
Patients with moderate-to-severe average pain, n (%)	9 (18)	19 (38)	.04	0 (0)	1 (2)	>.99	0 (0)	1 (2)	>.99
Secondary outcomes									
NRS^a^ score, mean (SD)									
Worst pain	6.4 (1.1)	6.1 (0.7)	.58	5.3 (0.9)	5.2 (0.7)	.40	4.7 (1.1)	4.7 (0.6)	.80
Least pain	2.8 (0.8)	3.3 (0.7)	<.001	2.2 (0.8)	2.7 (0.5)	<.001	1.7 (0.7)	2.2 (0.4)	<.001
Average pain	3.7 (0.9)	4.7 (0.6)	<.001	3.3 (0.8)	3.6 (0.5)	.01	2.5 (0.8)	3.1 (0.5)	<.001
Patients reporting pain trends, n (%)			.005			.83			.03
Decreasing	11 (22)	2 (4)		35 (70)	34 (68)		41 (82)	30 (60)	
Increasing	20 (40)	34 (68)		0 (0)	0 (0)		0 (0)	0 (0)	
Unchanged	19 (38)	14 (28)		15 (30)	16 (32)		9 (18)	20 (40)	
Pain interference with daily functioning (0-10 scale), mean (SD)									
General activities	4.3 (1.0)	5.0 (1.5)	.004	3.1 (0.7)	3.7 (1.3)	.009	2.6 (1.3)	2.5 (0.7)	.69
Mood	4.3 (2.4)	3.8 (1.7)	.30	2.4 (0.8)	2.8 (2.1)	.19	1.8 (1.2)	1.7 (0.7)	.65
Walking	9.9 (0.4)	9.9 (0.3)	.72	8.8 (0.9)	8.9 (0.6)	.52	7.4 (1.4)	8.2 (0.7)	<.001
Sleeping	3.9 (1.8)	4.1 (1.3)	.50	2.3 (1.0)	2.8 (0.7)	.11	1.3 (0.8)	2.2 (0.5)	<.001
Relationship with others	1.9 (1.2)	2.3 (1.0)	.14	1.3 (1.0)	1.5 (0.6)	.39	0.7 (0.8)	1.0 (0.4)	<.001
Enjoyment of life	2.4 (1.1)	2.7 (1.0)	.09	1.6 (0.9)	1.8 (0.5)	.12	1.0 (0.01)	1.2 (0.4)	.01
Cumulative consumption of hydromorphone (μg/kg/d)	43.9 (8.3)	38.7 (7.0)	<.001	33.1 (10.8)	32.8 (6.8)	.86	28.7 (9.6)	27.6 (6.2)	.53
Patients with analgesic-related complications, n (%)			.23			>.99			>.99
Postoperative nausea and vomiting	29 (58)	21 (42)		5 (10)	4 (8)		0 (0)	0 (0)	
Other	0 (0)	0 (0)		0 (0)	0 (0)		0 (0)	0 (0)	

a NRS: numeric rating scale.

### Secondary Outcomes

Pain experiences—including pain intensity, treatment, analgesic consumption, and complications—and their impact on patients’ daily lives are summarized in Table 2. Specifically, the intensity of least pain was significantly lower in group 1 versus group 2 on each of the 3 consecutive days: POD1 (mean 2.8, SD 0.8 vs mean 3.3, SD 0.7*; P*<.001), POD2 (mean 2.2, SD 0.8 vs mean 2.7, SD 0.5; *P*<.001), and POD3 (mean 1.7, SD 0.7 vs mean 2.2, SD 0.4; *P*<.001). Similarly, average pain was lower in group 1 versus group 2 across the 3 consecutive days: POD1 (mean 3.7, SD 0.9 vs mean 4.7, SD 0.6; *P*<.001), POD2 (mean 3.3, SD 0.8 vs mean 3.6, SD 0.5; *P*=.01), and POD3 (mean 2.5, SD 0.8 vs mean 3.1, SD 0.5; *P*<.001).

By contrast, no differences were observed in the intensity of worst pain between the groups. Although NRS scores for worst, least, and average pain decreased significantly over time in both groups, a significantly higher proportion of patients in group 1 than in group 2 reported pain relief on POD1 (n=11, 22% vs n=2, 4%*; P*=.005) and POD 3 (n=41, 82% vs n=30, 60%; *P*=.03). The impact of pain on daily activities was significantly lower in group 1 than in group 2 on POD1 (mean 4.3, SD 1.0 vs mean 5.0, SD 1.5; *P*=.004) and POD2 (mean 3.1, SD 0.7 vs mean 3.7, SD 1.3; *P*=.009). No significant between-group differences were observed in the impact of pain on mood, walking, sleeping, relationships with others, and enjoyment of life, except on POD3. Cumulative hydromorphone consumption on POD1 was significantly higher in group 1 than in group 2 (mean 43.9, SD 8.3 vs mean 38.7, SD 7.0; *P*<.001).

Patient self-efficacy for postoperative pain was evaluated using the PSES (Table 3). No significant differences were observed between the 2 groups prior to surgery; however, the PSES score was significantly higher in group 1 than in group 2 on POD3 (mean 29.3, SD 7.5 vs mean 25.2, SD 8.7; *P*=.01). No significant differences were found between the 2 groups in patient satisfaction on POD3 (mean 8.0, SD 1.6 vs mean 7.7, SD 1.9; *P*=.40) or in the duration of the hospital stay (mean 8.4, SD 0.97 vs mean 8.1, SD 0.89 days; *P*=.11). To explore the correlation between pain self-efficacy and pain severity, a univariate logistic regression analysis was performed. The incidence of moderate-to-severe pain was not significantly associated with the PSES score (*P*=.59). No differences were detected in the incidence of analgesic-related complications across the 3 PODs. The recorded adverse events consisted primarily of postoperative nausea and vomiting (Table 2), all of which resolved spontaneously.

**Table 3.** Patient Self-Efficacy Scale scores in postoperative pain assessment.

	Group 1	Group 2	*P* value
Presurgery, mean (SD)	26.8 (7.8)	24.4 (7.7)	.12
Postoperative day 3, mean (SD)	29.3 (7.5)	25.2 (8.7)	.01

## Discussion

### Principal Findings

Overall, this study demonstrated that the number of patients with moderate-to-severe average pain on POD1 after thoracic surgery in pediatric patients was significantly lower in group 1 than in group 2 following the implementation of SMS-based education (n=9, 18% vs n=19, 38%). Furthermore, both the lowest and average NRS scores decreased over the first 3 PODs, and a greater proportion of patients in group 1 than in group 2 reported a trend of decreasing pain (n=11, 22% vs n=2, 4%). Collectively, these findings indicate that the implementation of SMS-based education led to an improvement in postoperative pain management in pediatric patients. Notably, group 1 not only exhibited lower absolute NRS scores than group 2, but these scores also reflected a clinically relevant difference in pain severity: group 1 experienced mild pain, whereas group 2 experienced moderate pain (mean 3.7, SD 0.9 vs mean 4.7, SD 0.6). Together with the primary outcome showing that significantly more patients in group 2 experienced moderate-to-severe average pain, these improvements in pain experience are clinically meaningful. This interpretation is further supported by the significantly reduced impact of pain on general activities observed in group 1.

Group 1 demonstrated a higher cumulative consumption of analgesics on POD1, indicating that this group received more rescue analgesic doses through PCAP. This finding may partly explain the observed improvement in postoperative pain control in participants. Owing to the limited time available for preoperative education and the difficulty in effectively explaining or retaining detailed information, most patients and parents have an incomplete understanding of pain assessment and the goals of pain management [9,10,32]. Moreover, concerns regarding potential side effects often lead parents to be overly cautious about analgesic administration [11,33,34], resulting in a tendency to encourage children to tolerate pain rather than to administer analgesics proactively. Consequently, even with the PCAP, pain relief in pediatric patients may remain inadequate [5,35]. By contrast, parents and patients in group 1 were able to consult mobile push messages at any time and receive ongoing educational support when questions arose. This access facilitated more accurate assessment of pain intensity and timely administration of rescue analgesic doses in accordance with medical staff expectations.

Pain self-efficacy may represent another important determinant of pain experienced in this study. Although evidence regarding the impact of pain self-efficacy on postoperative pain in pediatric patients remains limited, studies in adults have demonstrated that enhancing pain self-efficacy can reduce postoperative pain intensity [36] and improve pain-related outcomes and patients’ overall recovery quality [37]. Moreover, studies conducted in pediatric patients with chronic pain have consistently reported beneficial effects of pain self-efficacy on children’s pain and quality of life [38-40]. Patients who are more confident in their pain management strategies typically experience less pain and distress. Accordingly, the lower pain intensity and improved general activity observed in group 1 may, at least in part, be attributable to enhanced pain self-efficacy, which is consistent with the findings of previous studies. However, this study did not detect a causal relationship between pain self-efficacy and pain severity. This may reflect the multifactorial nature of pain and be related to the relatively small sample size. Consequently, pain self-efficacy could not be confirmed as a variable independently associated with pain severity in this study. Further studies are warranted to clarify the underlying mechanisms.

Finally, pain is a highly subjective experience influenced not only by pharmacological and nonpharmacological analgesic strategies for analgesia but also by various other factors [41]. Previous studies have reported that placebo effects can alleviate pain by enhancing patients’ expectations of beneficial outcomes [42]. In this study, given the ubiquity of mobile phone use, the participants in group 1 could easily access relevant information anytime and anywhere, which may have strengthened their expectations of effective pain control. Moreover, because parents assume primary responsibility for postoperative pain management in children, their cognition, attitudes toward pain, and psychological states—such as helplessness, stress, depression, or confidence—inevitably influence children’s perceptions of pain. Parental responses to children’s pain and their sense of security have been shown to affect pediatric pain experiences. Accordingly, the observed changes in pain experience in this study may be partly attributable to the sense of security experienced by both parents and children, knowing that guidance and support were readily accessible through SMS.

Although general activity was significantly better in group 1 than in group 2, no significant between-group differences were observed in other domains of pain interference. Although group 1 reported better sleep, social functioning, and enjoyment of life compared with group 2 on POD3, scores in both groups remained at a satisfactorily low level. Therefore, further studies are warranted to determine whether these differences have a significant impact on clinical outcomes despite more favorable subjective evaluations. In addition, the highest NRS scores in both groups remained above 4 points over the 3 PODs, with no significant between-group differences. Similarly, walking ability scores in both groups remained above 5 points during this period, although group 1 performed significantly better than group 2 on POD3. These findings suggest that pain during movement was inadequately controlled in both groups. This limitation may explain the absence of a significant difference in overall patient satisfaction despite improvements in average pain intensity and general activity in group 1. It may also be related to the relatively low analgesic consumption observed among study participants compared with that reported in previous studies [43,44]. Further studies are needed to optimize pain management and achieve more effective postoperative analgesia in patients.

Overall, the data indicate that implementing SMS for postoperative pain management in pediatric patients undergoing thoracic surgery significantly enhanced pain self-efficacy, effectively reduced pain intensity, and improved daily functioning. These findings indicate the importance of patients’ subjective experiences in clinical practice and highlight the role of interventions that increase patient engagement and confidence in pain self-management. This study provides a framework for progressing interventions from feasible and acceptable to effective. Given the limitations of existing research, future studies should examine a broader range of clinical scenarios and more diverse patient populations. This could include exploring different modalities (eg, SMS, mobile apps, and web-based platforms), varying disease contexts (eg, chronic pain, cancer, and postoperative pain), different care settings (eg, hospital and home), and relevant comorbidities (eg, depression and anxiety) to validate findings and enhance generalizability. Such efforts would support the translation of research findings and facilitate the clinical implementation of high-quality SMS interventions for pain management in pediatric populations.

### Study Limitations

This study has some limitations that should be considered when interpreting the results. First, the study was conducted at a single center, the Children’s Hospital of Fudan University, one of the largest pediatric medical centers in an economically developed region of China. Parents of patients at this center generally have a higher socioeconomic status, better level of education, and greater compliance with medical directives. Consequently, the single-center design and relatively small sample size may limit the generalizability of the findings. Second, the study focused solely on the impact of SMS text messages on postoperative acute pain management in pediatric patients classified as ASA physical status I or II. Inclusion of patients with ASA physical status III or higher could have broadened the applicability of the results. However, at the Children’s Hospital of Fudan University, the pediatric patients were transferred to the intensive care unit, where more frequent and detailed pain assessments and interventions are performed by medical staff. Such intensive management would inevitably influence patients’ self-management and the study outcomes, thereby justifying the exclusion of patients with more severe conditions.

Second, there is a significant concomitant relationship between pain and psychiatric disorders, with complex interactions. For example, pediatric patients with attention-deficit/hyperactivity disorder are more prone to pain [45], which may be related to increased pain sensitivity [46]. Pain and pain-related dysfunction may be further exacerbated in patients with depression and anxiety [47], while patients with autism spectrum disorders may exhibit abnormal pain perception [48]. Thus, pain management for pediatric patients with comorbid psychiatric disorders is a distinct and complex topic; however, it was not the focus of this study. Therefore, children with psychiatric disorders were excluded from this study. Owing to variations in pain characteristics and patient populations, the results obtained in this specific cohort may not be generalizable to broader populations. However, the findings can serve as a reference for calculating sample sizes in future studies.

Third, self-reporting bias is another consideration. Pain, the primary outcome, was assessed using self-reported measures, which may be influenced by memory errors, social desirability bias, or comprehension differences. Randomization was used during the study design to ensure balanced baseline characteristics and minimize selection bias. Although participants were not blinded to group allocation, data collectors were blinded, reducing the risk of researcher expectation bias and its potential impact on participants’ reported outcomes. During the data collection phase, validated standardized questionnaires and scales were used to quantify the participants’ self-reported results, ensuring consistency in data collection and reducing differences in subjective interpretation. Furthermore, pain experience was evaluated from multiple perspectives. In addition to the primary outcome of moderate-to-severe pain incidence, secondary assessments included quantified pain scores, daily pain intensity trends, pain-related functional impairment, and quality of life measures, such as general activity, mood, and sleep. Continuous follow-up over 3 PODs enabled a comprehensive assessment across multiple dimensions and reduced reliance on single self-reports. Participants also received thorough education regarding research objectives and reporting procedures, thereby enhancing compliance and mitigating single-report bias. However, reporting bias could not be entirely eliminated.

Finally, the study did not investigate the underlying causes of pain and/or contributing factors, including individual pain perception, nonpharmacological strategies, psychological status, or the interaction of analgesic use and pain experience, which limited insight into the mechanisms influencing the observed outcomes.

### Conclusions

The implementation of SMS-based postoperative pain management in pediatric patients undergoing thoracic surgery improved clinical outcomes. However, its effects on patient outcomes and the underlying mechanisms require further investigation.

## Supplementary material

10.2196/81806 Multimedia Appendix 1 Example of the educational information sent to participants in group 1.

10.2196/81806 Multimedia Appendix 2 Detailed version of the information sent to participants, using self-assessment methods for pain (translated to English).

10.2196/81806 Checklist 1 CONSORT-eHEALTH checklist (V1.6.1).

## Abbreviations

ASA: American Society of Anesthesiologists
BPI: Brief Pain Inventory
NRS: numeric rating scale
PCAP: patient-controlled analgesia pump
POD: postoperative day
PSES: Pain Self-Efficacy Scale
