# Supplementary figures and images for: The Effect of Text Messaging on the Postoperative Pain Experience in Pediatric Patients Undergoing Thoracic Surgery: Randomized Controlled Trial
Source: J Med Internet Res. 2026 Mar 24;28:e81806. doi: 10.2196/81806 (PMC13012229; doi:10.2196/81806)

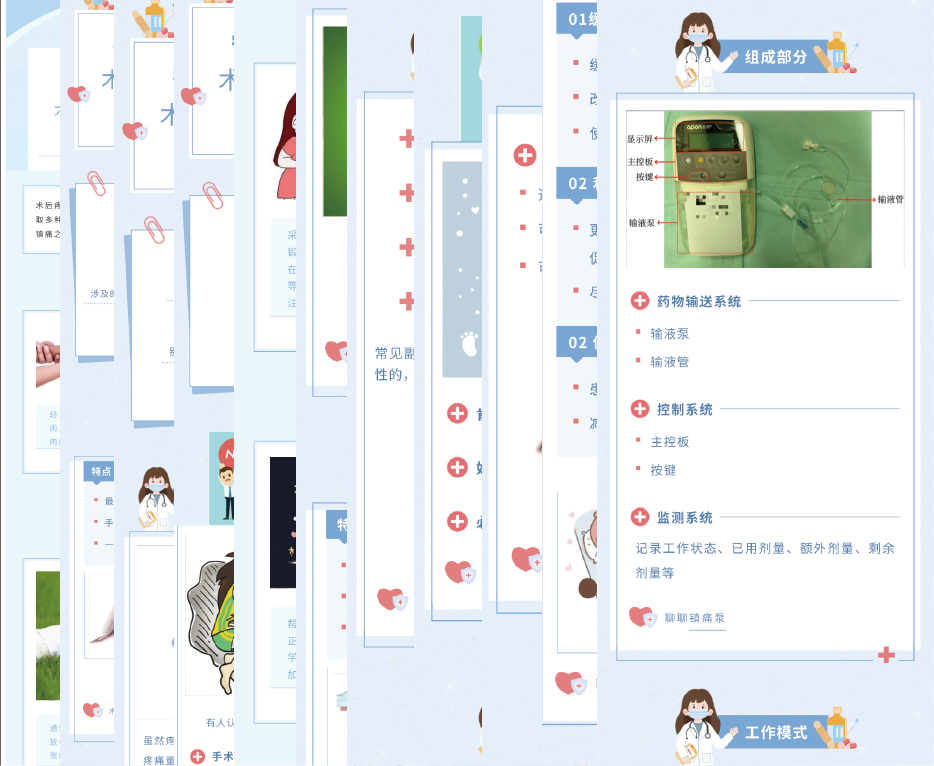

Supplement: Multimedia Appendix 1 [file jmir-v28-e81806-s001.png]

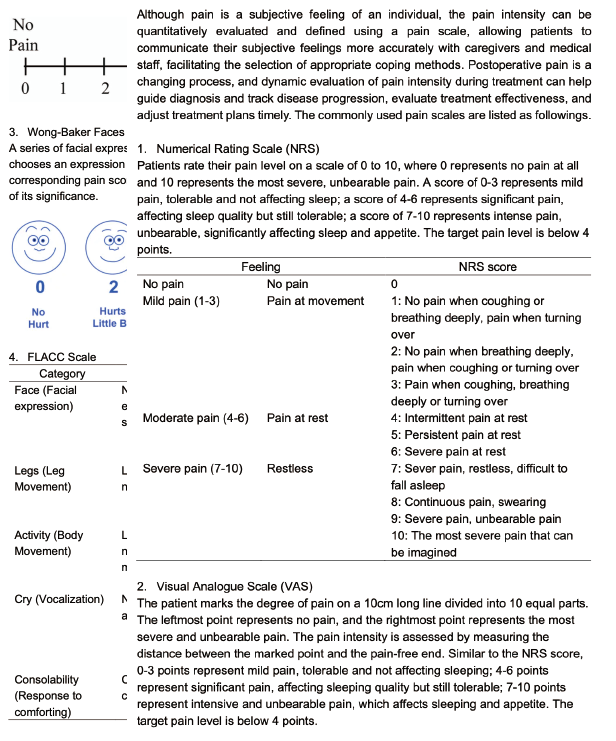

Supplement: Multimedia Appendix 2 [file jmir-v28-e81806-s002.png]
